# The Search for the Ideal Female Breast: A Nationally Representative United-States-Census Study

**DOI:** 10.1007/s00266-021-02753-y

**Published:** 2022-01-18

**Authors:** Christoph Wallner, Vanessa Dahlmann, Paolo Montemurro, Sherko Kümmel, Mattea Reinisch, Marius Drysch, Sonja Verena Schmidt, Felix Reinkemeier, Julika Huber, Johannes Maximilian Wagner, Alexander Sogorski, Mehran Dadras, Maxi von Glinski, Marcus Lehnhardt, Björn Behr

**Affiliations:** 1 Department of Plastic Surgery, BG University Hospital Bergmannsheil, Ruhr University Bochum, Bürkle-de-la-Camp Platz 1, 44789 Bochum, Germany; 2 Akademikliniken, Storängsvägen 10, 11541 Stockholm, Sweden; 3 Breast Center, Essen-Mitte Clinics, Henricistr. 92, 45136 Essen, Germany

**Keywords:** Ideal breast, Morphometry, Female

## Abstract

**Introduction:**

Many studies have started to search for the perfect aesthetic breast in order to create a pars-pro-toto for reconstruction, but especially for aesthetic surgery. To date, no representative study with anatomically accurate models was performed.

**Methods:**

In an online based United-States-census-representative survey with 1049 participants, questions regarding the preferred breast were asked utilizing lifelike morphed 3D-generated female models for the first time. Attributes such as breast pole ratio, areola size, breast direction and projection were asked.

**Results:**

The results show that, contrary to what has been claimed in previous studies, an upper-pole-to-lower-pole ratio of 55:45 is preferred by both female and male participants. When it comes to breast size, on the other hand, there are clear gender-specific differences. While women opted for a cup size around B, the men preferred larger cup sizes. Moreover, the smallest depicted areola size of 30 mm was favored among all groups in the survey.

**Discussion:**

Most publications used rather detrimental models for their surveys. We therefore opted for computer-generated 3D models and varied their naturalness. This enabled us to ensure a more aesthetic and accurate illustration and thus obtained more comparable and reliable results paired with the representation of the US-population. Taken together this study unveiled unexpected insights into the population favored breast attributes that might change operative planning in breast surgery.

**Level of Evidence V:**

This journal requires that authors assign a level of evidence to each article. For a full description of these Evidence-Based Medicine ratings, please refer to the Table of Contents or the online Instructions to Authors http://www.springer.com/00266.

## Introduction

Over time, the optical ideal of the female breast has changed and there are also different preferences in different cultures. Today's preference is mainly shaped by the increased use of media and social media recently. The breast stands as a symbol for femininity, not only in its designation as a secondary gender characteristic, but also in society. Many women identify themselves as women through them, and the aesthetics of the breast contribute significantly to their well-being. With over 287,000 breast augmentations in 2019 it is one of the most performed surgical procedures in the USA [1]. Whether it is breast enlargement or reduction for purely aesthetic reasons, as a wish in the context of transsexuality, or breast reconstruction after oncological interventions, the demand remains high. A not inconsiderable proportion of women who have had breast surgery tend to repeat operations in search of improved or satisfactory results [2, 3]. Several studies have already dealt with the question of what the ideal woman's breast looks like, but in the past, however, they focused on isolated parameters [4, 5].

So far, not conclusive anatomical model has been reported

The aim of this study is to create a model for the ideal breast with an optimized model in combination with a population-based survey that can be utilized to optimize reconstructive and aesthetic breast surgery.

## Methods

### Study design

A web-based questionnaire with several sub-categories was created depicting the most discussed elements in aesthetic breast surgery. The first part contained general questions about demography, level of education, relationship status as well as the feeling about the own breast, the own body preference as well as the attitude towards aesthetic interventions. The second part contained sets of different series of images, each with a morphed property. The questionnaire was entered into the SurveyMonkey (San Mateo, USA) system. The target audience was reached through SurveyMonkey® Audience with a guarantee of a cross national sample. To represent the US-population, a sample size was chosen that has a margin of error of 4% with a confidence level of 99%.

The following parameter were separately morphed into the image panels: Proportions of upper and lower pole (35:65, 40:60, 45:55, 50:50, 55:45) in two different body types, the size of the mamilla (30 mm, 35 mm, 40 mm, 45 mm, 50 mm), the upper and lower pole fullness (100%, 50%, 0%, − 50%, − 100%) in the three-quarters profile view as well as side view, the distance from the median line to the mamilla (105 mm, 110 mm, 115 mm, 120 mm, 130 mm) in the three-quarters profile view as well as front view, a nine images panel with different characters of fullness and natural profiles, different cup sizes of the breast (AA, A, B, C, D, E) in the three-quarters profile view as well as front view.

3D models were generated and morphed in DAZ Studio 3D (version: 4.12.1.117; DAZ 3D, Inc., Salt Lake City, USA). Morphed pictures were randomly shuffled for each respondent to avoid a bias.

### Statistical analysis

After testing for homo- or heteroscedasticity (*F*-Test), *p* values for pairwise comparisons were analyzed via two-tailed unpaired *t* test with (heteroscedasticity) or without (homoscedasticity) Welch’s correction. For correlation between two bivariate nominal values Phi and Cramer-V test were performed for two bivariate ordinal Kendall-Tau-B. All analyses were performed using GraphPad PRISM (version: 8.3.0; Graphpad Software, Inc., California, USA). Statistical significances were set at a *p* value < 0.05.

## Results

### Survey population

A total of 1049 participants from the USA completed the survey through the platform SurveyMonkey. According to the age and gender the participants reflect the US-census with a margin of error of ± 3.91%. The median time to complete the survey was 5:06 minutes with a completion rate of 95%. Most of the participants were Caucasian (67%), followed by Asian/Pacific Islander (10%), Hispanics (10%), Black/African Americans (9%), multiple ethnicities and others (2%) and American Indian or Alaskan Natives (2%). 922 (88%) respondents were born in the USA. Further details are depicted in Table 1.

**Table 1:** Overview of the national representative survey population

	Value
Age (yrs)	
18–24	143 (14%)
25–34	237 (23%)
35–44	186 (18%)
45–54	166 (16%)
55–64	159 (15%)
65–74	123 (12%)
> 75	35 (3%)
Sex	
Male	554 (53%)
Female	486 (46%)
Transsexual	8 (1%)
Other	1 (0.1%)
Ethnicity	
American Indian or Alaskan Native	19 (2%)
Asian/Pacific Islander	108 (10%)
Black/African American	95 (9%)
Hispanic	105 (10%)
White/Caucasian	700 (67%)
Multiple Ethnicity/Other	22 (2%)

### Breasts with a ratio of 55:45 were most popular

**Fig. 1:**
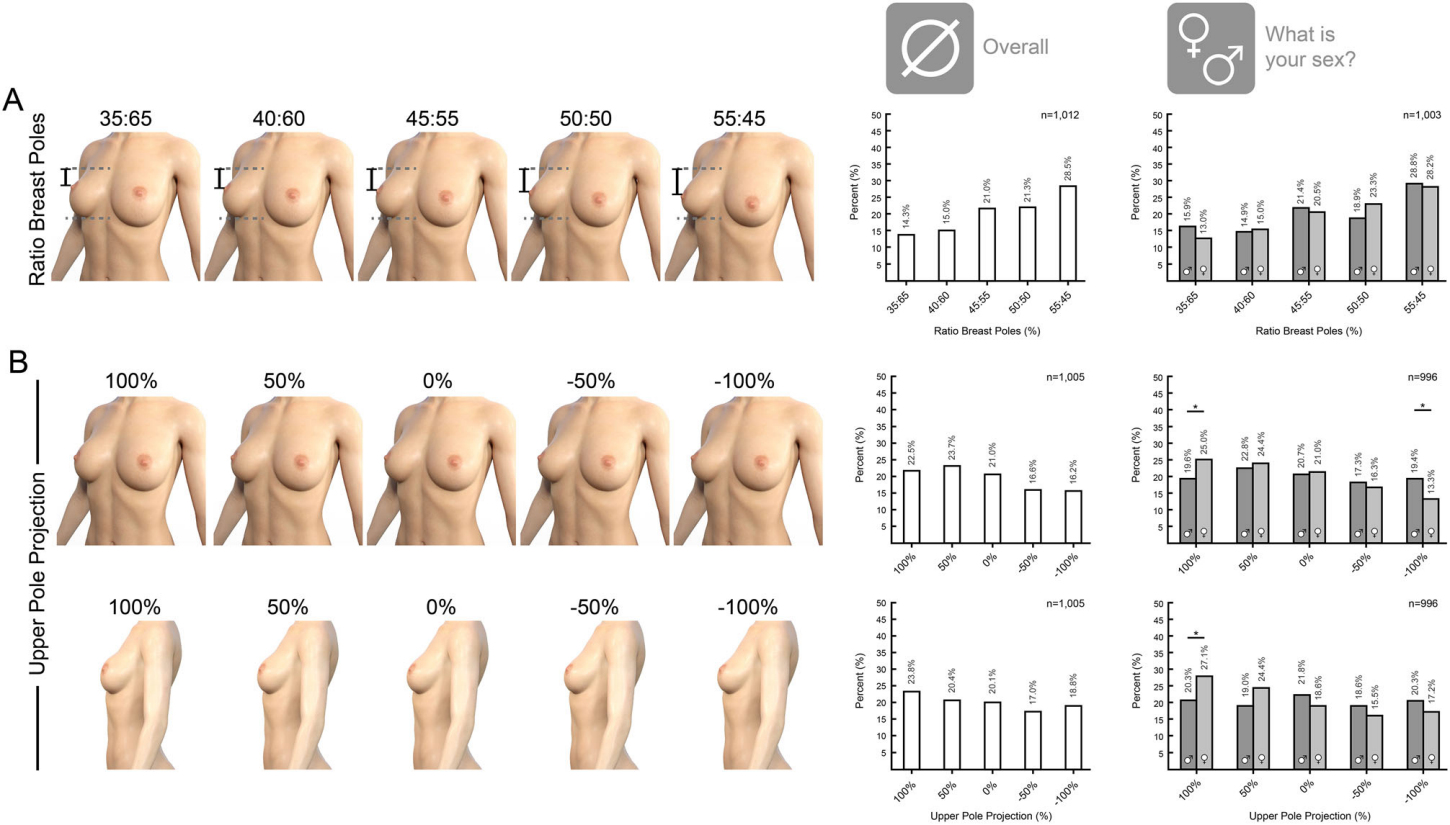
Survey results on the preferred breast ratio and projection. **A** In the survey 1012 participants responded to the question what ratio of the breast poles is preferred. 532 female and 471 male participants responded. With 28.5% the majority of the overall population prefer a ratio of 55:45 between the upper and the lower pole. Differentiating between male and female participants, there is no change in this trend upon the subgroups. **B** In the survey 996 participants responded to the question what percentage of the upper pole projection compared to the initial image is preferred. 528 female and 468 male participants responded. In the ¾-view there is an overall trend towards upper pole projection. There is a divergence in preference between men and women in both the ¾-view and the side-view. Women significantly prefer an increased upper pole projection, while men show no preference. This diverging correlation is significant in both views (*p* = 0.05). For correlation between two bivariate nominal values Phi and Cramer-V test were performed. For comparing two single values two-sample t-test was performed. *p* value: * < 0.05

1012 participants responded to the question “Please select the most aesthetic breast from the following pictures?” with different ratios of the breast poles. There is an overall tendency toward a small lower pole. Around 50% of all participants preferred 50:50 and 55:45 ratios. Distinguishing female and male respondents showed no difference in breast pole ratio preferences. In the second question the favored upper pole projection was determined by 1005 participants. In DAZ Studio 3D the upper pole projection was changed according to the program settings (-100% to +100% upper pole projection). Overall, the participants preferred an increased upper pole projection. However, female participants exceptionally selected enhanced upper pole projection in the ¾-view and side view. This trend was statistically significant comparing women and men (*p* = 0.05)—see Fig. 1.

### Small areolae and narrow breasts were favored

**Fig. 2:**
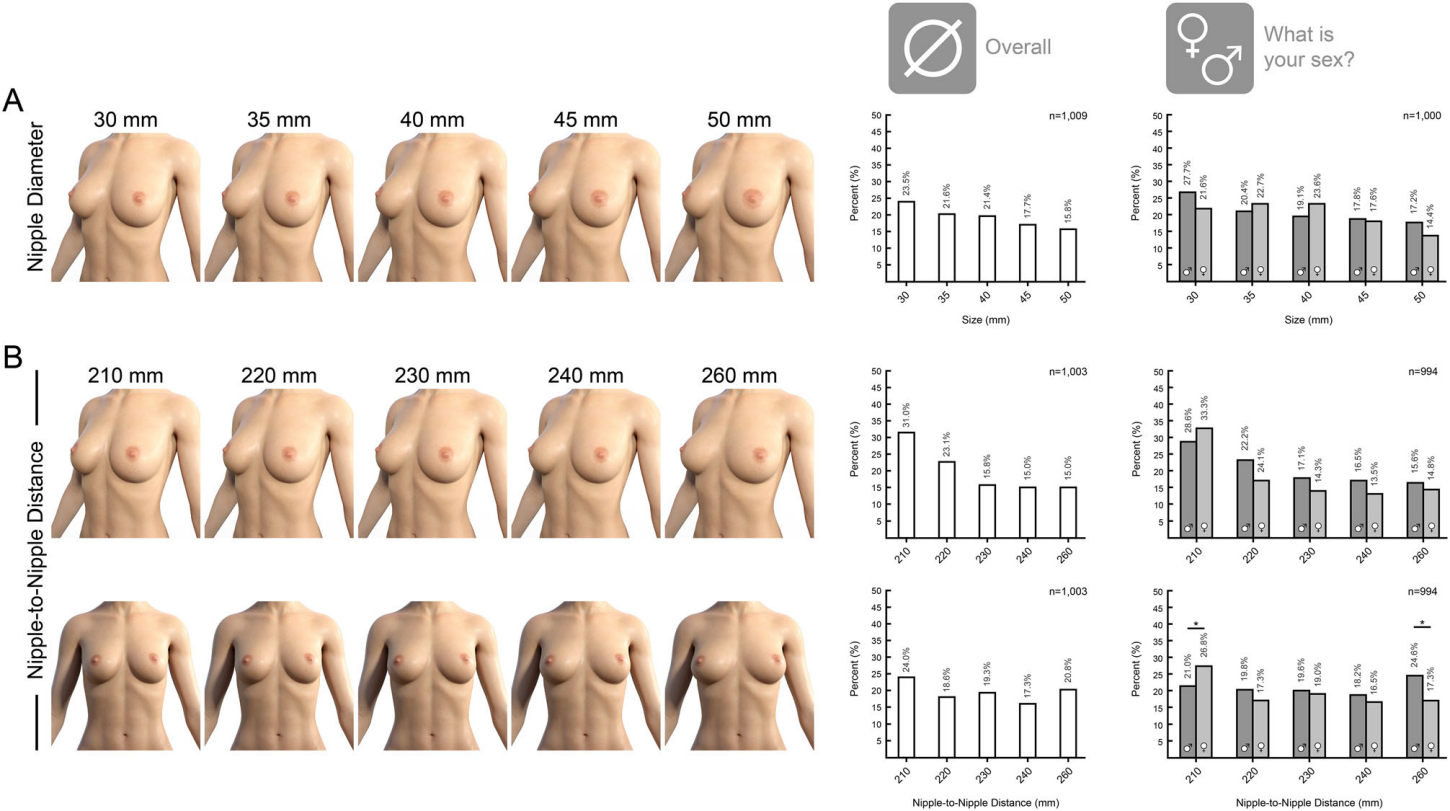
Survey results on preferred areola diameter and nipple-to-nipple distance. **A** In the survey 1000 participants responded to the question what areola diameter is preferred. 529 female and 471 male participants responded. With 23.5% the majority of the overall population prefer the smallest areola-size of 30 mm. With larger areola the preference decreases. Differentiating between male and female participants, there is no change in this trend upon the subgroups. **B** In the survey 994 participants responded to the question what distance between the nipples is preferred. 526 female and 468 male participants responded. In the ¾-view there is an overall trend towards narrow breasts with 210 mm distance. There is a divergence in preference between men and women in the frontal view. Women prefer a significantly narrower breast while men show no specific preference. This diverging correlation is significant in the frontal view (*p* = 0.03). For correlation between two bivariate nominal values Phi and Cramer-V test were performed for two bivariate ordinal Kendall-Tau-B. For comparing two single values two-sample t-test was performed. *p* value * < 0.05

The areola diameter was accurately transferred onto the 3D models with Measure Metrics for DAZ Studio (version: 1.8.0.2; DAZ 3D, Inc., Salt Lake City, USA). Overall, 1009 participants preferred smaller areolaes. There was no significant difference comparing female and male responses. In another question the preference of the nipple-to-nipple distance was determined among 1003 participants. Taken together, a preference for narrow breasts with the shortest in the survey covered distance of 210 mm was mostly selected. This trend was attenuated in the front view, connoting a different perception of the diameter in the different view. In both views, male participants tended to a longer nipple-to-nipple distance with a significant correlation in the frontal view as compared to women (*p* = 0.03)—see Fig. 2.

### Men preferred larger breasts than women

**Fig. 3:**
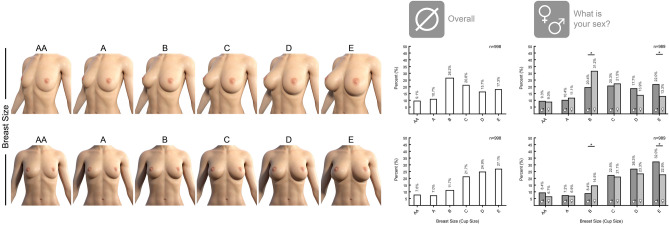
Survey results on preferred breast cup size. **A** In the survey 989 participants responded to the question what breast cup size is preferred. The breast cup sizes were calculated by the difference between bust and underbust circumference according to the EN 13402. 525 female and 464 male participants responded. In the 3/4 -view breasts larger than A cups are preferred. The vast majority of the female participants preferred B-cup breasts. Differentiating between male and female participants, there is general tendency for men to prefer larger breasts than women. This trend is also shown in the frontal view. This diverging correlation is significant both in the ¾-view (*p* < 0.001) and the frontal view (*p* = 0.004). For correlation between two bivariate nominal values Phi and Cramer-V test were performed. For comparing two single values two-sample t-test was performed. *p* value: * < 0.05

Another question series addressed the preferred breast cup size. Breast size was adjusted to the model by applying the EN 13402 defined cup sizes under the guidance of Measure Metrics for DAZ Studio (version: 1.8.0.2; DAZ 3D, Inc., Salt Lake City, USA). In the 3/4-view the overall population (*n* = 998) favored B and C cup sizes. Breaking down the sex of the participants, male tended to choose larger breasts while women prefer B-cup in the 3/4-view. This disparity was statistically significant (*p* < 0.001) comparing women and men. In the frontal view there was an overall trend to larger breast sizes. However, the tendency of men to favor larger breasts was conserved and statistically significant (*p* = 0.004)—see Fig. 3.

### How does a previous breast augmentation or the desire for a breast augmentation influence breast attribute preferences?

In the survey, female participants (*n* = 554) were asked whether they had a previous breast augmentation or never had any cosmetic surgery done on their body. Participants were excluded who already had any other cosmetic procedure done. Excluding those a total of 425 female participants responded to this question. Both groups were compared regarding their responses about the favored breast cup size. Once a woman received a breast augmentation, she favored larger breasts. This was significant in the 3/4-view (*p* = 0.05) as well as in the frontal view (*p* = 0.026) comparing women with previous breast augmentation and women without any cosmetic procedure in the history. The mean actual cup size of women in both groups was a C-cup.

Furthermore, the preference for breast size was compared between women who consider breast augmentation and women who would not have their breasts enlarged. Participants who consider breast surgery due to aesthetic reasons tend to prefer larger breast sizes in the 3/4-view (*p* < 0.001) and front view (*p* = 0.03) as well. The mean actual cup size of women in both cohorts was a C-cup.

**Fig. 4:**
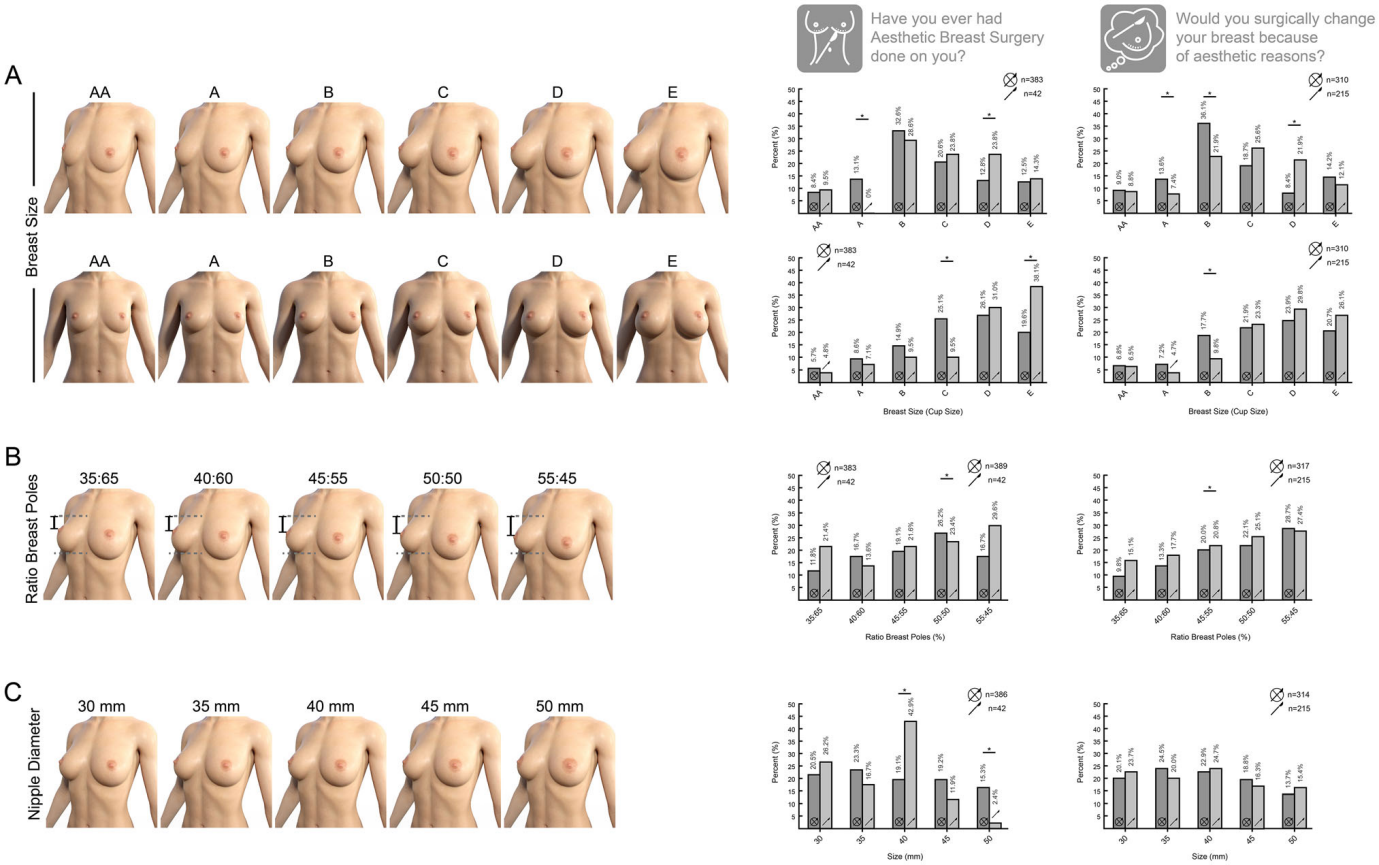
Survey results comparing the groups of participants who had aesthetic surgery done on their breast and participants who would like to change their breast surgically of aesthetic reasons. **A** Two groups of totally 425 participants were compared based on their response to the question of whether they had ever had aesthetic breast surgery or never had any aesthetic procedure done. The breast cup sizes were calculated by the difference between bust and underbust circumference according to the EN 13402. The response to the preferred breast cup size showed a significant tendency towards larger breasts in participants who had pervious breast augmentation. This correlation was shown in 3/4 -view (*p* = 0.05) as well as in the frontal view (*p* = 0.026). For the question whether the female participants would surgically change their breast because of aesthetic reasons 525 responses were collected. Participants who would want surgically change their breast because of aesthetic reasons tend to prefer larger breast sizes in the ¾-view (*p* < 0.001) and front view (*p* = 0.03). **B** The same groups were compared for their response to the preferable ratio of the breast poles. There was no statistically significant correlation detectable in either group. **C** The same groups were compared for their response to the preferable areola diameter. Female participants who had surgically augmented their breast prefer smaller areola diameter (*p* = 0.003), while there is no significant correlation between the question whether they would surgically change their breast because of aesthetic reasons and the areola diameter preference. For correlation between two bivariate nominal values Phi and Cramer-V test were performed for two bivariate ordinal Kendall-Tau-B. For comparing two single values two-sample t-test was performed. *p* value: * < 0.05

Asking the same groups for their preferences in breast pole ratios there was no significant correlation. Women who had a prior breasts augmentation performed, prefer smaller areola diameters (*p* = 0.003) compared to participants who never had not received prior cosmetic breast procedure. This correlation was not observed between participants who would change their breast size and who would not—see Fig. 4.

## Discussion

The aim of this study was to define the perfect female breast with its various features in its global aesthetic but also to validate the results of previous studies. With the help of this definition, breast surgery should be improved, and the satisfaction of the women operated on should be increased.

A study from 2016 came to the conclusion that the natural-looking breast with an upper pole-to-lower-pole ratio of 45:55 percent is perceived as the most attractive [4]. Due to the few parameters and the way the survey was carried out with Photoshop-manipulated images which appeared unnatural the results are questionable (a standard at that time). The same authors identified four key features of the aesthetic breast through a study consisting of 100 topless cover models. The different models show a subjectively appealing breast, but questions remain unanswered: Who selected the women, who is the target group of these media and ultimately the breasts vary from model to model in several parameters. In our study, these weaknesses could be eliminated. On the one hand, only one parameter is changed in every picture-series in the 3D models, and on the other hand, a completely new range of target group results when a representative of the entire population of a country is surveyed.

Another study emphasizes that in order to find the ideal breast, all breast features must be taken into account [5]. The distribution of preferences clearly in favor of 45:55 in the mentioned publication underlines the invalidity of alternatives edited in Photoshop, due to their appearing unnaturalness. A normal distribution is completely absent in this particular study demonstrating a lack of the methodology [4]. Our study with anatomical accurately manipulated breasts shows a Gaussian distribution that supports the validity of the results. Other studies are not able to reproduce this feature.

In contrast to all previous studies, more breast characteristics are considered simultaneously in order to determine the ideal breast in its entirety. A 3D model that can be shifted in its naturalness, as used in this study, enables comparable and valid results to be achieved. Many behavioral studies rely on schematic illustration of body proportions [6, 7]. However, with emerging technologies such as 3D computer graphics this field of research can be substantially enhanced. Today, it is possible to render an incredibly natural looking human and modify proportions without factitious looking deformities as it could arise in Photoshop manipulated images. With the new technology of realistic human 3D rendering, it was possible to achieve a harmonious breast despite manipulation of individual parameters in our study. An investigation of breast preference based on highly realistic breast representations has not yet been carried out and represents an extraordinary novelty in this study. Highly realistic 3D render should be used not only in plastic surgery but also in the representation of the human body as a whole for studies evaluating various changed characteristics such as the waist-to-hip ratio.

An absolute novelty is the finding that most of the survey participants, both female and male, preferred an upper-pole-to-lower-pole proportion of 55:45. In previous studies, the 45:55 proportion has been the most attractive [4]. This new finding could possibly be an US-American phenomenon. This argument is supported by the fact that an upper pole projection was also favored, depicting more unnatural breast attributes.

The two sexes disagreed slightly on the ideal diameter of the nipple. The average areolae around 30–40 mm were favored by women. Men were more in favor of a smaller areolae of 30 mm. A small areola was found to symbolize youthfulness as it grows with maturation and pregnancies [8]. According to a study by Dixson et al. men’s preferences for women’s areolar size appear to be highly culturally specific. Dixson et al. stated the areolar pigmentation as an additional strong signal for maturation of the breast [9]. This feature was not included in this study.

We found a clear difference in the preference for breast size between women and men. While women were more interested in medium-sized breasts, around breast size B, larger cup sizes were favored by men. Given that large breasts exude femininity and fertility, larger sizes might be an important atavistic selection criteria for men [10]. Studies with eye-tracking have shown, that men spend more time on looking at breasts and upper-body than any other region underlining this female attribute in male perception of female attractiveness [11]. This is highly conserved in different cultures including indigenous people without access to modern media [9]. The difference between diverging male and female perception of the ideal breast might arose from the runaway sexual selection which is the most widely-accepted theory about development of the female breast as a sign of nulliparity, age, or sexual maturity [12].

The preference for upper pole projection was also quite different. Women opted for an upper pole projection of 100%. The reason for this could be that a full filled breast is a sign of youth and the loss of the upper pole projection is a result of the aging process [8]. The male participants in the study did not speak out in favor of any specific type, as did the nipple-to-nipple distance, where the female participants chose a narrow distance of around 21cm.

We see partially unexpected results in the study, such as the preference for smaller areoles but also the distribution of the breast at 55:45. On the contrary, there is a normal distribution around these results, which in turn speaks for a range of preferences. A conclusion for our clinical work should be the consideration of the individual preference and not the adherence to given numbers. Nevertheless, there are tendencies such as the small areole size but also the breast distribution that can be implemented in clinical practice.

A high veritability of our study can be seen from the age distribution of the study participants. This forms a kind of Gaussian bell curve and thus seems to be a very good representation of the population. The study depicts the US-census a margin error of 3.91%. This fact sets our study apart from all previous ones. Other studies either have subjectively selected models that vary in too many parameters (size, distribution, body weight, ethnic background, skin quality, areola size, foot print, etc.) or the target audience is biased, e.g., by a panel of experts or selected target groups, e.g., patients who have undergone aesthetic surgery. As a result, the present study is the only one of its kind to represent the population adequately.

Female participants who underwent or consider to breast augmentation tend to prefer larger cup sizes. However, neither of the groups differ significantly in their actual cup sizes. One confounder of this finding might be the age groups. There were significantly more participants with breast augmentation prorated in the group of the 35 to 44 years old women compared to women without any aesthetic intervention in the same age group. Older female participants had prorated fewer breast augmentations in the group of 65 years old women and above. On the other hand, there was no significant correlation between the age group and a preference for the cup-size overall. Interestingly female participants who underwent a breast augmentation prefer smaller areola sizes but not the group of participants who would do an augmentation. Again, there is no significant correlation between age groups of female participants and the preference for a specific areola size. While women around the age of 25 to 44 tend to be more open to aesthetic surgery and underwent more aesthetic procedures there is no specific age dependent preference for the breast cup size or areolar diameter. The reason for this finding might be a sociocultural and medial bias on participants who had or tend to do aesthetic breast surgery. They are influenced by (social) media, more informed and might have higher expectations on their breast as these participants follow-up with the look of their breast.

The main limitation of our study are the different views of the models. When evaluating our results, a difference was found between front view and 3/4-view. This is particularly evident in the breast size and the nipple distance. This incongruence is probably due to possible light shadows that arise from the change of perspective in the images. The finding of divergent perceptions due to differing views is a current obstacle in psychological research [12]. However, our findings for the preference for larger breasts in more realistic models are supported by Dixson and Zelazniewicz. They also report these realistic models as a more ecologically valid stimulus [11, 12].

Another point worth discussing is the representation of different ethnic groups in the models. Nevertheless, the model was limited to one ethnic group, as including more ethnicities would have made the survey unmanageably complex and therefore lead to many survey cancellations. In addition, there is the question of depicting the breast shapes of different ethnic groups, for which there is too little data or simply changing the skin color, which would not have added any added value. Therefore, limitation to one ethnic group as most of the previous studies was the most suitable option.

**Fig. 5:**
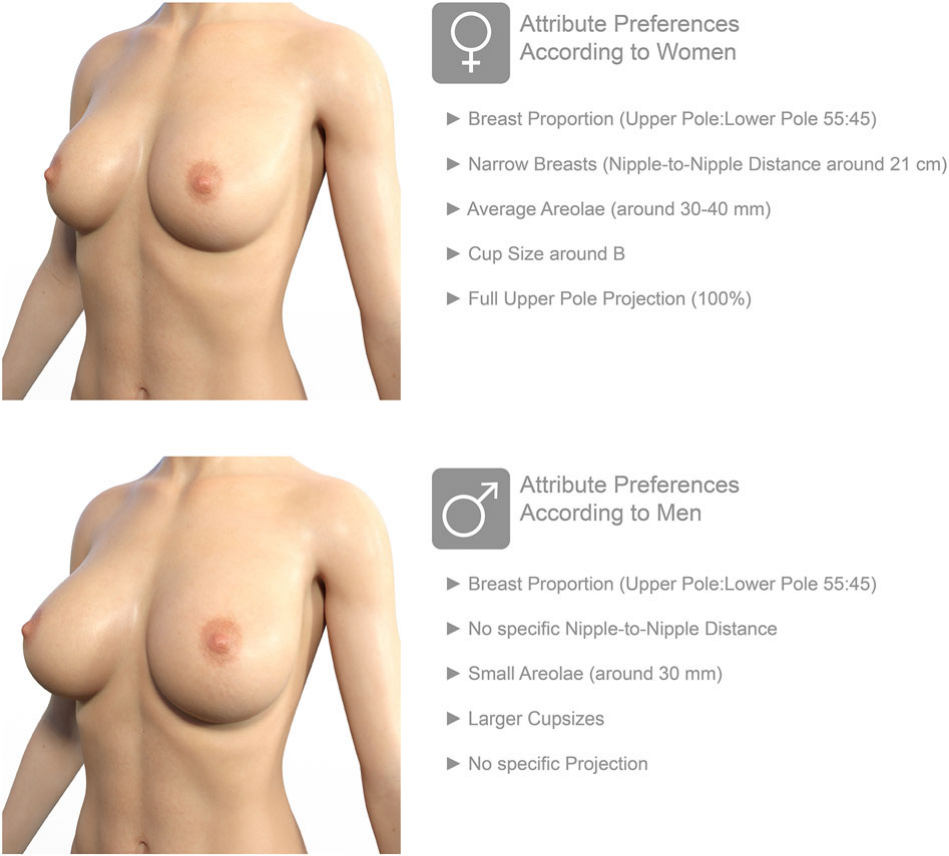
Illustration and overview of attribute preferences in breast according to men and women

The summarized findings of this study are taken together in Fig. 5 with features incorporated in 3D models.

## Conclusion

Based on the methodological composition of the study through the census-based survey with over 1000 participants and the naturally morphed 3D models, we conclude a realistic representation of the preference for the female breast. The purpose of the study is not to set up one-sided guide values for proportionality or nipple size, but rather to do away with dogmatic, sometimes invalid, statements. The study provides tendencies, but a key statement of the study remains the individuality of each breast, which is not subject to a given value. Further aspects of this study that should be incorporated into clinical practice are breast augmentation that has already taken place, changes the patient's expectations, but also the fact that social media has an increasingly important influence on self-image.

In summary, it can be stated that especially the newly discovered upper-pole-to-lower-pole-proportion of 55:45 and the areola size of 30 mm are remarkable and could influence future breast surgery.
